# 

**Published:** 1918-03-30

**Authors:** 


### CONTENTS.

| | PAGE |
|---|---|
| **Psychological Tests or Rubbish?** ... ... | 541 |
| **The Territorial Medical Officers...** ... ... | 542 |
| **Hospital and Institutional News ...** ... ... | 543 |
| A Financial Policy for Leeds General Infirmary—Applying the Policy all Round—Mr. Hodge on Charles Dickens—Officers and Hospital Stoppages—The Brighton Pavilion Hospital's Library—Educational Lectures for the Wounded—The Iniquities of the Voting System—The Opportunity at Kingston—The Extensions at Ealing Hospital—Complaints Against a Medical Board—The Reasons for the Classifications—A Drastic Reconstruction Demanded—The Seamen's Hospital, Marseilles—A New Children's Hospital—The Gift of a Meal—The Organisation of Outlying Districts—The Fight Against Anthrax—The Standardisation of Salvarsan Substitutes—Food and the Invalid—The Third London *Gazette*—To our Readers. | |
| **The Museum as a Teaching Centre** ... ... | 547 |
| Bone-Grafting in Relation to Sepsis. | |
| **The Mental Deficiency Act at Work** ... ... | 548 |
| A Surprising War-Time Achievement. | |
| **Lord Lister** ... ... ... ... ... ... | 549 |
| X. His Search for Three Ideals. | |
| **Some Annual Meetings** ... ... ... ... ... | 550 |
| **A Ministry of Health** ... ... ... ... | 551 |
| Medical Treatment in the Home. | |
| **Parliament and the Profession** ... ... ... | 552 |
| **Physical Culture in Education** ... ... ... | 553 |
| II. The Problem of the Town-bred Child. | |
| **Nursing Affairs in Ireland ...** ... ... ... | 554 |
| Propaganda: The Effort Towards Union. | |
| **The Matrons' and Sisters' Department** ... | 555 |
| Nursing Progress and its Developments: XI. The Grandeur of the Nation's Nursing Services. | |
| Round the Hospitals. | |
| Eastertide 1918—The Nation and the Armies' Ordeal—The War-work of Nurses—The Barnsley Hospital Inquiry Begun—County Hospital, Colchester: Inquiry Demanded—A Nurses' Roll of Honour. | |
| **Matrons' and Sisters' Appointments** ... ... | 558 |
| **The College of Nursing** ... ... ... ... | 559 |
| Meeting of the Liverpool Centre. | |
| **The Nation's Fund for Nurses** ... ... ... | 559 |
| Meeting at Plymouth. | |
| **Mr. G. Q. Roberts on St. Thomas's Hospital History** ... ... ... ... ... ... | 560 |
| A Few of his Interesting Photographs. | |
| **The British Hospitals Association** ... ... | iii |
| The Annual Meeting. | |
| **The Institutional Worker ...** ... | (Suppl.) 1 |
| Sugar Beet: Its Cultivation and Use. | |
| March Questions. | |
| Questions Invited. | |

